# CD19-specific triplebody SPM-1 engages NK and γδ T cells for rapid and efficient lysis of malignant B-lymphoid cells

**DOI:** 10.18632/oncotarget.13110

**Published:** 2016-11-04

**Authors:** Christian B. Schiller, Todd A. Braciak, Nadja C. Fenn, Ursula J. E. Seidel, Claudia C. Roskopf, Sarah Wildenhain, Annemarie Honegger, Ingo A. Schubert, Alexandra Schele, Kerstin Lämmermann, Georg H. Fey, Uwe Jacob, Peter Lang, Karl-Peter Hopfner, Fuat S. Oduncu

**Affiliations:** ^1^ Department of Biochemistry and Gene Center, Ludwig-Maximilians-University, Munich, Germany; ^2^ Division of Hematology and Oncology, Medizinische Klinik und Poliklinik IV, Klinikum der Universität München, Munich, Germany; ^3^ Department of General Paediatrics, Oncology/Haematology, University Children's Hospital Tübingen, Tübingen, Germany; ^4^ Department of Biochemistry, University of Zurich, Zurich, Switzerland; ^5^ Department of Biology, University of Erlangen-Nuremberg, Erlangen, Germany; ^6^ Westend-Innovation, Munich, Germany

**Keywords:** single chain triplebody, antibody-dependent cellular cytotoxicity, gamma delta T cell, leukemia, immunotherapy

## Abstract

Triplebodies are antibody-derived recombinant proteins carrying 3 antigen-binding domains in a single polypeptide chain. Triplebody SPM-1 was designed for lysis of CD19-bearing malignant B-lymphoid cells through the engagement of CD16-expressing cytolytic effectors, including NK and γδ T cells.

SPM-1 is an optimized version of triplebody ds(19-16-19) and includes humanization, disulfide stabilization and the removal of potentially immunogenic sequences. A three-step chromatographic procedure yielded 1.7 - 5.5 mg of purified, monomeric protein per liter of culture medium. In cytolysis assays with NK cell effectors, SPM-1 mediated potent lysis of cancer-derived B cell lines and primary cells from patients with various B-lymphoid malignancies, which surpassed the ADCC activity of the therapeutic antibody Rituximab. EC_50_-values ranged from 3 to 86 pM. Finally, in an impedance-based assay, SPM-1 mediated a particularly rapid lysis of CD19-bearing target cells by engaging and activating both primary and expanded human γδ T cells from healthy donors as effectors.

These data establish SPM-1 as a useful tool for a kinetic analysis of the cytolytic reactions mediated by γδ T and NK cells and as an agent deserving further development towards clinical use for the treatment of B-lymphoid malignancies.

## INTRODUCTION

CD19 is a type I transmembrane glycoprotein and a signaling receptor of the immunoglobulin superfamily. It is a component of the B cell receptor (BCR) co-complex, and is expressed from early to late stages of B cell development. Signaling through CD19 promotes proliferation and differentiation [1–4]. The antigen is particularly promising for targeted immunotherapy of B-lymphoid malignancies, because of its relatively high and lineage-specific expression on B cells of different maturation stages and its presence on the surface of cancer progenitor cells [5–7]. However, the results of initial clinical trials with CD19-antibodies were disappointing [5, 8], presumably due at least in part to a far lower surface density of CD19 on the malignant cells than of CD20 on lymphoma cells, the target antigen for the clinically successful antibody Rituximab.

A second generation of improved CD19-targeting proteins and cellular agents has been developed, which includes Fc- and glyco-engineered immunoglobulins, such as the antibodies XmAb5574 (MOR208) and MEDI-551 [9, 10], the bispecific T cell engager (BiTE^®^) Blinatumomab (Blincyto®) [11, 12], and the adoptive transfer of genetically modified T cells, obtained by stable transfection with chimeric antigen receptors (CARs). Genetically modified CAR-T cells (CAR-Ts) have been remarkably successful in the treatment of various B-lymphoid malignancies, in particular of Acute Lymphoblastic Leukemia (ALL) in children and young adults with poor prognosis [13–16]. CD19 therefore clearly is a useful target for antigen-specific immunotherapy, provided an appropriate molecular format of the therapeutic agent is chosen.

BiTE and CAR-T cell approaches rely on cytotoxic T lymphocytes (CTLs) for the elimination of cancer cells [11, 13–16]. Although highly effective in many cases, these approaches still leave room for future improvements. In rare cases, undesirable adverse events have been reported after treatment of patients with CD19-directed BiTEs and CD19-directed CAR-Ts. These include cytokine release syndromes and involvement of the central nervous system [17] as well as tumor lysis syndrome and the occasional outgrowth of target antigen-negative tumor cell clones (“antigen-loss” or “escape” variants) [12, 17, 18]. A short serum half-life in the case of BiTEs, potentially durable lymphopenia in the case of CARTs and finally, the associated high costs limit the availability of these therapies for a significant segment of patients.

The use of antibodies for therapeutic purposes, on the other hand, is well-established and effective, and is generally less expensive, but also suffers from certain limitations. These agents rely on the presence of Fc-receptor-bearing effector cells, which can be activated by the mediator protein to assemble a productive synapse with the target cell. In some cases, the needed effector cells are not available in sufficient quantity and quality at certain stages of disease development. An example are limiting numbers of functionally active NK cells in the bone marrow of AML patients at diagnosis, which limit the use of IgGs as front-line therapeutic agents [19–22]. Immunoglobulins further suffer from limited penetration into solid tumor tissues, owed to their large mass [23–25].

To address some of these limitations of mono-targeting IgGs, our team has developed the molecular format of single chain triplebodies. These proteins consist of 3 single chain variable antibody fragments (scFvs) connected by flexible (G_4_S)_n_ linkers [26–31]. Triplebodies are anticipated to have improved pharmacokinetic properties due to their smaller size of approximately 90 kD. Based on experimental and theoretical data from other groups, this is expected to result in a faster equilibration of these molecules in the mammalian blood circulation and in an easier penetration into solid tumor tissues compared to full-length antibodies [23, 32, 33]. Furthermore, triplebody SPM-2 was shown to mediate an efficient elimination of AML blasts from an AML patient in remission by autologous NK cells [34].

Triplebodies developed so far were designed for bivalent binding of a tumor cell via their two distal scFvs, and for monovalent binding to a trigger molecule on a cytolytic effector cell through their central scFv. Effector cells engaged by triplebodies to date include NK cells, CTLs, and neutrophilic granulocytes, which were activated for cytolysis via the triggers CD16, CD3ε, and CD64, respectively [35]. CD16, the human Fcγ-receptor III (FcγRIII), is expressed by NK cells, macrophages, γδ T cells and a subset of cytokine-stimulated dendritic cells (DCs). Upon cross-linking, signaling via CD16, which associates with the FcR γ-chain or CD3ζ, elicits potent effector functions including ADCC and phagocytosis [36].

NK cells and γδ T cells are capable of recognizing and eliminating malignant cells in a natural killing mode in the absence of mediator proteins such as antibodies. NK cells respond to the absence of MHC class I proteins on cancer cells (“missing self”). γδ T cells are independent of MHC : peptide recognition, but can react to non-peptidic phosphoantigens, which are preferentially displayed on the surface of cancer cells. Therefore, both of these effector cell classes can bypass immune evasion of cancer cells by downregulation of MHC class I [37, 38]. In addition, NK cells and γδ T cells are major anti-viral effectors after transplantation of hematopoietic stem cells (HSCT), but do not cause graft-versus-host-disease (GvHD) [37]. Enhancing the cytolytic activity of NK cells and γδ T cells via CD16-triggering therapeutic agents therefore is a promising strategy for cancer treatment, which may also lead to a systemic cellular immune response following an initial lysis of cancer cells by ADCC, in the sense of a tumor-vaccination effect.

The capacity to recruit γδ T cells as cytolytic effectors has not yet been established for triplebodies triggering through CD16, although it had been reported for other agents in related molecular formats, including the Fc-engineered CD19-antibody 4G7SDIE and the bispecific CD19-targeting fusion protein N19-C16 [39–41]. Therefore, we anticipated that a similar result may also be obtained for corresponding triplebodies, but experimental verification of this hypothesis was still needed. These agents have different sizes and space-filling properties, and may therefore differ in their steric access to CD16 epitopes on NK and γδ T cells. To test this hypothesis, the previously described triplebody ds(19-16-19) [26], with 2 binding sites for CD19 and one for CD16, was humanized and stabilized, and the new agent was named SPM-1. The protein was purified, characterized and compared here in NK cell-based ADCC assays to other CD19-specific agents in related molecular formats. In support of the hypothesis, SPM-1 was found capable indeed of mediating lysis of CD19-bearing target cells in combination with human γδ T cells as effectors in ADCC assays. In these studies we also observed that SPM-1 mediated a particularly rapid lysis of substrate-bound CD19-bearing target cells in a time-resolved, impedance-based assay, the XCelligence^®^ assay [41, 42]. This stimulating finding calls for additional future studies of the kinetics of the ADCC process mediated by antibody-derived agents with different molecular architecture, which will likely lead to a better understanding of the unusually high cytolytic potential of triplebodies compared to some of the related bi-specific agents [26].

## RESULTS

### Design and production of triplebody SPM-1

**Figure 1:**
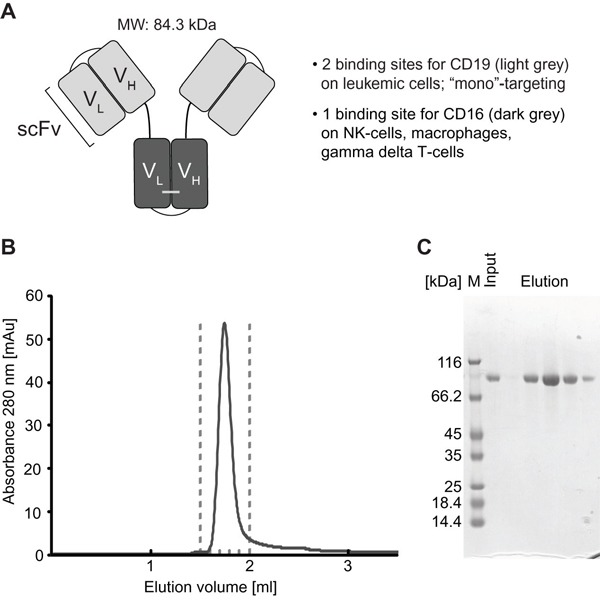
Structural characteristics and purification of triplebody SPM-1 **A.** CD19-specific scFv domains shown in light grey; CD16-specific scFv domain in dark grey. **B.** After purification with the 3-step chromatographic procedure described in Methods, SPM-1 eluted in size exclusion chromatography profiles (SEC) as a single monodisperse peak corresponding to the molecular mass of an SPM-1 monomer. Species of higher molecular mass (aggregates) were absent/below detection limit. **C.** Fractions from the SEC elution profile (eluting between the dashed lines in B) were analyzed by SDS-PAGE and stained with Coomassie blue. The preparation was of high purity; higher and lower molecular mass species were absent or only very minor contaminants.

Triplebody SPM-1 was constructed based on the published parental triplebody ds(19-16-19) [26]. The scFv domains of the parental protein were derived from the murine monoclonal antibodies 4G7 and 3G8. To minimize immunogenicity, the CD16- and CD19-directed scFv sequences were humanized by CDR grafting [43, 44] plus subsequent adjustment of the framework. The humanized CD16 scFv was then disulfide-stabilized, whereas the 2 humanized CD19-binding domains were used without further stabilization, because this protein was sufficiently stable compared with disulfide-stabilized variants, which have also been studied (Figure 1A and additional data not shown). The cDNA coding sequences for the 2 CD19-specific scFvs were adjusted by mutations in the wobble bases to minimize homologous recombination in the producer cells, and thus to improve production yields of the desired protein. This procedure reduced the occurrence of truncated variants, which had been observed prior to this optimization.

SPM-1 was expressed in Freestyle™ 293-F cells (Life Technologies) and was purified in a 3-step procedure, which included capture from culture supernatants by zinc ion affinity chromatography followed by anion and cation exchange chromatography. This capture step and the ion exchange chromatography protocol followed industry standard procedures, and the downstream purification process is scalable and suitable for commercial production. Yields ranged from 1.7 - 5.5 mg of purified protein/L of culture supernatant in different production runs (Table 1). Purified SPM-1 eluted as a single monomeric peak in size exclusion chromatography profiles and was highly pure as evidenced by SDS-polyacrylamide gel electrophoresis (Figure 1B, 1C).

**Table 1:** Biological activity and antigen affinity of triplebody SPM-1

Construct	Yield [mg/L cell culture]	Biol. Activity (SEM) EC50 [pM]	Affinity CD16 [nM]	Avidity CD19 [nM]
**SPM-1**	1.7 - 5.5	11 ± 3	12.0 ±1.5	17.5 ± 0.4

The affinity is the equilibrium binding constant K_D_ for monovalent binding to CD16 on stably transfected CHO-CD16 cells; the avidity is the K_D_ value of the molecule for bivalent binding to CD19-bearing SEM target cells.

### Binding characteristics of SPM-1

Specificity of SPM-1 binding to CD19 and CD16 was established by cytofluorimetry with suitable control antibodies and control cells (unpublished data). The monovalent affinity of SPM-1 for CD16 and the bivalent avidity for CD19 were measured by determining the equilibrium binding constants (K_D_) by flow cytometry with CD16- or CD19-bearing human cells as previously described [45, 46]. The K_D_ value of SPM-1 for CD16 was 12.0 ± 1.5 nM, which was lower than the value of 58.6 ± 4 nM reported for the parental triplebody ds(19-16-19). Therefore, the humanized plus disulfide-stabilized CD16 binding domain carried in SPM-1 displayed 4- to 5-fold stronger binding to human CD16 on living cells than the corresponding domain of the parental protein. The K_D_ value of SPM-1 for CD19 (17.5 ± 0.4 nM) was comparable to the value measured for the parental protein (13.0 ± 1.2 nM; Table 1) [26], and therefore, the humanization procedure has not significantly altered the binding avidity of the second-generation triplebody for CD19.

### Cytolytic activity of SPM-1 in combination with human NK cells

**Figure 2:**
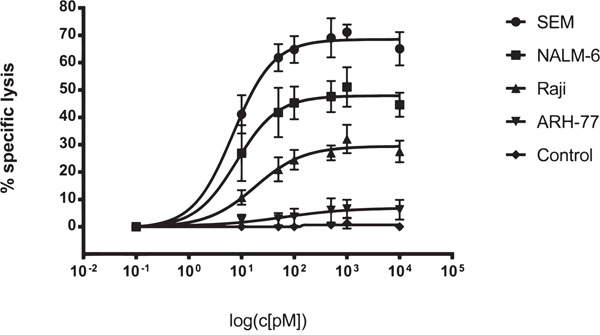
SPM-1 mediates lysis of a panel of CD19-bearing cell lines derived from various types of B cell malignancies Dose response profiles from Redirected Lysis (RDL) assays performed with SPM-1 or control proteins plus *ex vivo* expanded MNCs from healthy donors. Calcein release assays as described in Methods. SEM cells were derived from a pro-B ALL, NALM-6 from a pre-B ALL, RAJI from a Burkitt's Lymphoma, and ARH-77 from a multiple myeloma. MNCs were used at an 8 : 1 effector-to-target cell (E : T) ratio, corresponding to a net NK : target cell ratio of 2 : 1, because NK cells accounted for approx. 25 % of the expanded MNC population. SPM-1 concentrations in the reaction mixtures in pM units. Specific lysis plotted on the vertical axis was computed as explained in Methods. The control triplebody targeting HER2 failed to induce specific lysis at comparable concentrations as SPM-1, because this antigen was undetectable on the target cells used here. In combination with HER2-bearing targets this triplebody was active in positive control experiments, performed separately. Data points plotted here are mean specific lysis percentages ± standard error of the mean (SEM) from n = 4 to 5 separate experiments.

The ability of SPM-1 to mediate cytolysis of human cancer cells by NK cells from unrelated healthy donors was tested in standardized redirected lysis (RDL) assays against malignant human B-lymphoid cell lines at an effector-to-target cell (E : T) ratio of 2 : 1. The target cell lines represented different classes of B-lymphoid neoplasias and carried different mean surface densities of CD19 (Table 2). All tested cell lines displayed a dose-dependent cytolytic response to treatment with SPM-1 plus NK cells (Figure 2). The potential of these cell lines for specific lysis was weakly correlated with their mean target antigen density: the SEM line with the highest CD19 density displayed the strongest cytolytic response, whereas the ARH-77 line with the lowest density displayed the weakest response. No specific lysis was observed, when the specificity control triplebody Her2-16-Her2 was used (Figure 2), as expected, because Her2 is not expressed on these target cells. The measured EC_50_-values of 5.6 pM for the SEM line and 79.4 pM for ARH-77 cells (Table 1) were in the same range as the values previously reported for the non-humanized parental triplebody, which were 4.1 and 29 pM, respectively [26]. Therefore, in combination with NK cells from an unrelated healthy donor, SPM-1 displayed overall similar cytolytic potential for malignant B-lymphoid cell lines as the parental triplebody.

**Table 2:** Surface expression of CD19 and EC_50_ values for RDL by SPM-1 with different malignant B lymphoid cell lines as targets

Cell Line	Subtype of malignancy	CD19 density [#]	EC_50_ (range) [pM]
SEM	Pro-B (mixed lineage)	42,600 ± 10,700	5.6 (2.5 – 10)
NALM-6	Pre-B	26,700 ± 11,400	6.1 (1.8 – 17.3)
RAJI	Burkitt's Lymphoma	31,900 ± 22,800	16.5 (5.4 – 38.5)
ARH-77	Mature B (myeloma)	2,400 ± 1,200	79.4 (38 – 645.3)

Antigen densities are given in copy numbers per cell.

### SPM-1 mediates stronger lysis of some primary cancer cell samples by NK cells than the therapeutic antibody Rituximab (MabThera®)

**Figure 3:**
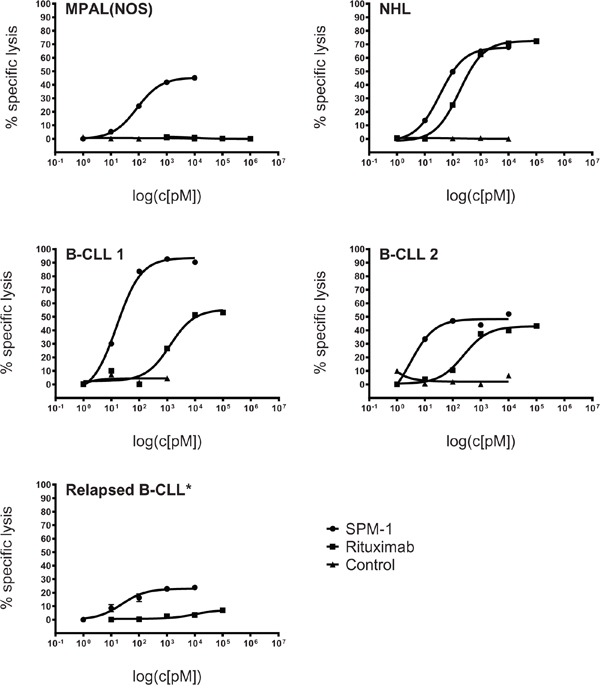
SPM-1 mediates stronger lysis of primary lymphoma- and leukemia blasts from newly diagnosed patients than the therapeutic antibody Rituximab (MabThera®) Malignant cells from peripheral blood of newly diagnosed patients were used as targets in RDL and ADCC assays with SPM-1 and Rituximab, respectively. SPM-1 and Rituximab were present in the reaction volumes in the concentrations shown in pM units. NK cells were used at a net E : T ratio of 2 : 1, as defined for Fig. 2. Samples were from 1 patient with a mixed phenotype acute leukemia (MPAL(NOS); CD19^+^ CD20^−^); 2 patients with B-CLL (CLL: chronic lymphocytic leukemia; CD19^+^ CD20^LOW^); 1 patient with a Non-Hogkin Lymphoma (NHL; CD19^+^ CD20^HIGH^) and 1 patient with newly relapsed B-CLL (CD19^+^ CD20^DIM^). This patient had undergone 6 previous treatments with Rituximab. Insufficient primary material from this patient was available to perform the HER2-16-HER2 control, which is indicated by an asterisk (*).

The ability of SPM-1 to mediate cytolysis of a panel of primary B-lymphoid cancer cell samples in conjunction with NK cells was compared with the corresponding ability of the clinically successful CD20 antibody Rituximab (MabThera®). Peripheral blood samples from 2 newly diagnosed B-CLL patients, from a relapsed B-CLL patient 4 years after treatment with Rituximab, from a Non-Hodgkin lymphoma patient with leukemic progression, and from an adolescent patient with a mixed phenotype acute leukemia (not otherwise specified) (MPAL (NOS)) were collected. Mean target antigen densities on the blast surfaces were determined by calibrated cytofluorimetry, and all blast populations with exception of the MPAL (NOS) sample were double-positive for CD19 and CD20 at varying mean densities (Table 3). The MPAL (NOS) sample was CD20-negative. The newly diagnosed B-CLL and NHL samples displayed dose-dependent responses to both SPM-1 and Rituximab, whereas the MPAL (NOS) sample responded only to treatment with SPM-1 (Figure 3). Blasts from the relapsed B-CLL patient did not respond to treatment with Rituximab under these conditions, although they expressed CD20 on their surface, and these cells were therefore not antigen-loss escape variants. They displayed a weak, but clearly measurable dose-dependent response to treatment with SPM-1. Therefore, they still were capable of responding to NK-mediated lysis, and thus their failure to respond to treatment with Rituximab must have been due to other causes. The EC_50_-values for SPM-1 were 5- to 430-fold lower than those for Rituximab under these experimental conditions (Table 3).

**Table 3:** Target antigen densities and EC_50_ values for RDL / ADCC by SPM-1 or rituximab with primary lymphoma and leukemia blasts isolated from newly diagnosed patients

Patient	Antigen density [#]		EC_50_ [pM]	
	CD19	CD20	SPM1	Rituximab
B-CLL 1 (at diagnosis)	9,600 ± 500	4,000 ± 100	15.8	247.5
B-CLL 2 (at diagnosis)	7,600 ± 1,900	3,900 ± 1,200	3.0	1,300
Relapsed B-CLL	6,500 ± 1,700	1,800 ± 300	27.0	-
MPAL(NOS)	8,400 ± 2,800	0	86.0	-
NHL	14,600 ± 7,700	19,400 ± 2,200	35.7	185.7

Antigen densities are given in copy numbers per cell.

### SPM-1 mediates comparable cytotolytic activity by NK cells as related Fc-engineered antibody-derived agents

**Figure 4:**
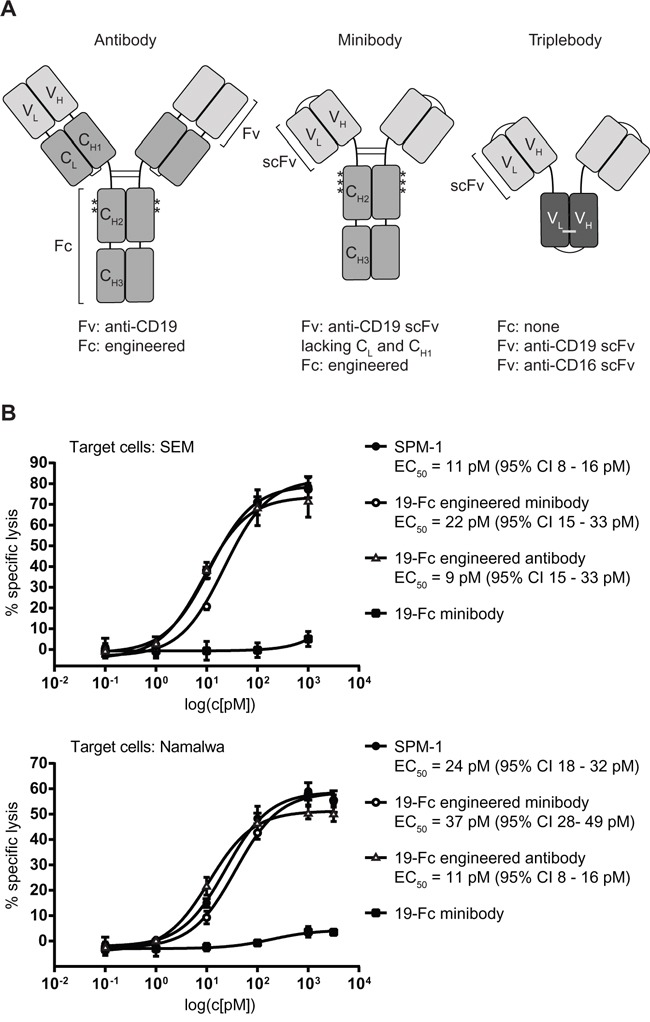
Triplebody SPM-1 performs equally well as other best-in-class CD19-specific agents in related molecular formats in comparative RDL/ADCC assays **A.** Asterisks indicate positions of point mutations (substitutions S239D and I332E) in the Fc region of the Fc-engineered antibody 4G7SDIE. Single chain fragment variable (scFv) units used in the minibody and the triplebody are labeled. One minibody carried the non-engineered Fc-domain, the other the same 2 mutations S239D and I332E shown above for 4G7 SDIE plus a third substitution A330L (third asterisk). **B.** RDL analysis of SPM-1 (filled black circles) compared with the best-in-class antibody 4G7SDIE (open triangles), the Fc-engineered minibody (open circles) and the non-engineered minibody (black squares). Target cells: SEM (top) and Namalwa (bottom).

After having established that triplebody SPM-1 was capable of mediating effective lysis of cancer cells via the CD16-specific scFv domain in ADCC experiments with human NK cells, we asked how strong this activity was compared with related molecular formats of antibody-derived proteins, which carry an Fc portion optimized for ADCC activity by suitable point mutations. To this effect we employed 2 CD19-specific minibodies, one with an engineered Fc-domain, mutated for optimized ADCC activity, the other with the non-mutated Fc-portion from the parental human IgG1 antibody. In addition, we had access to the Fc-engineered CD19-antibody 4G7SDIE [39, 41] for comparative cytotoxicity studies, which carried some of the same mutations in its Fc-portion as the optimized minibody (Figure 4A). Standardized 3 hr cytolysis assays were performed with NK cells from a healthy donor at an E : T ratio of 2 : 1 against SEM (pro-B ALL) and Namalwa (Burkitt lymphoma) target cells. No difference was observed between the dose-response of the target cells to treatment with SPM-1 or the Fc-engineered CD19 antibody 4G7SDIE and the Fc-engineered minibody, while treatment with the non-Fc-engineered minibody was ineffective (Figure 4B). Remarkably, SPM-1 and the Fc-engineered 4G7SDIE antibody produced a similar degree of maximum specific lysis in this endpoint assay, and both were active with similar EC_50_-values, while clear differences in the kinetics of their action in ADCC experiments with γδ T cells as effectors were observed in the impedance-based, time-resolved assays described below (Figure 6; Supplementary Figure S1). Therefore, release assays measuring only the endpoint of cytotoxicity integrated over an entire measurement interval, typically of 3 - 4 hrs, fail to reveal kinetic details of the reaction, which however are likely to be important for an understanding of the *in vivo* activity of these agents in animal models and human recipients.

### Activation of non-pre-stimulated γδ T cells from peripheral blood of healthy donors by SPM-1 plus target cells

**Figure 5:**
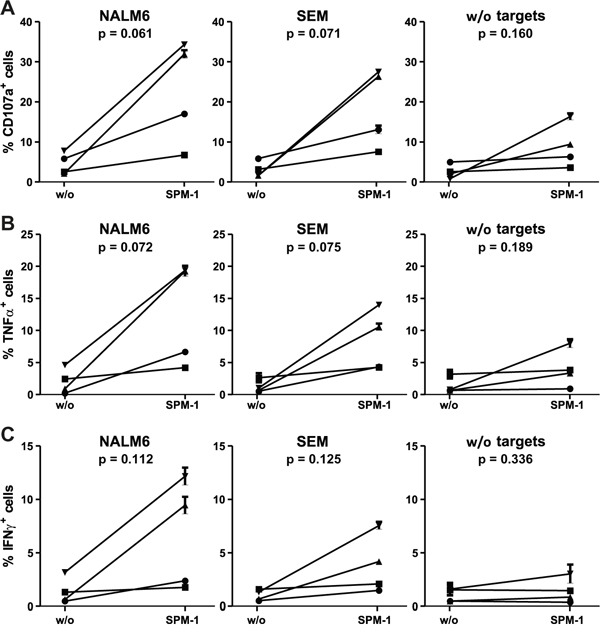
Primary γδ T cells are activated for cytolysis by exposure to SPM-1 plus target cells **A.** Fresh γδ T cells were prepared from healthy donors as described in Methods and exposed to SPM-1 with or without NALM-6 or SEM target cells. Degranulation of γδ T cells was monitored indirectly by cytofluorimetric measurement of the surface antigen density of the degranulation marker CD107a. **B.** Changes in intracellular TNFα concentrations in primary γδ T cells following exposure to SPM-1 plus/minus target cells were measured cytofluorimetrically as described in Methods. **C.** Changes in intracellular IFN-γ concentrations in primary γδ T cells occurring after exposure to SPM-1 plus/minus target cells were measured cytofluorimetrically as described in Methods. Of the 6 samples from different donors that were analyzed, 4 had a γδ T cell content above 2 % in their PBMC compartments and each of these responded to exposure to SPM-1 plus target cells by increased cytokine production and increased surface exposure of the degranulation marker in comparison to treatment with the control triplebody SPM-2 or exposure to the target cells alone without mediator proteins.

Human γδ T cells express CD16 and are capable of mediating ADCC of malignant targets in combination with CD19-antibodies and the antibody-derived bispecific agent N19-C16 [39–41]. Therefore here we asked, whether primary human γδ T cells from healthy donors, not pre-stimulated by other means, can be activated for cytolysis by exposure to SPM-1 plus target cells. Peripheral blood mononuclear cells (PBMCs) were isolated from platelet-pheresis products from healthy donors and the fraction of γδ T cells in this population was (2.3 ± 1.2) % on average. These low frequencies necessitated an indirect detection method for the activation of primary untreated γδ T cells. To this effect, CD19-bearing pro-B (SEM) and pre-B ALL (NALM-6) cells were first incubated overnight with primary samples of γδ T cells, and subsequently SPM-1 or control agents were added and ADCC activity was indirectly monitored by measuring the appearance of the degranulation marker CD107a on the surface of the γδ T cells. In addition, intracellular concentrations of TNFα and IFN-γ were measured by cytofluorimetry as markers for activation towards cytolysis. In the absence of CD19-bearing targets, γδ T cells of only a few sensitive donors displayed a weak elevation of CD107a on their surface and a small increase in intracellular TNFα after addition of SPM-1. However, after overnight incubation with CD19-bearing target cells, γδ T cells from several donors showed a clear increase in cell surface CD107a and intracellular TNFα and IFN-γ after addition of SPM-1, and thus showed clear evidence for activation towards cytolysis by SPM-1 plus target cells (Figure 5).

### SPM-1 mediates target cell lysis by both primary non-expanded and *ex vivo* expanded γδ T cells from healthy donors

**Figure 6:**
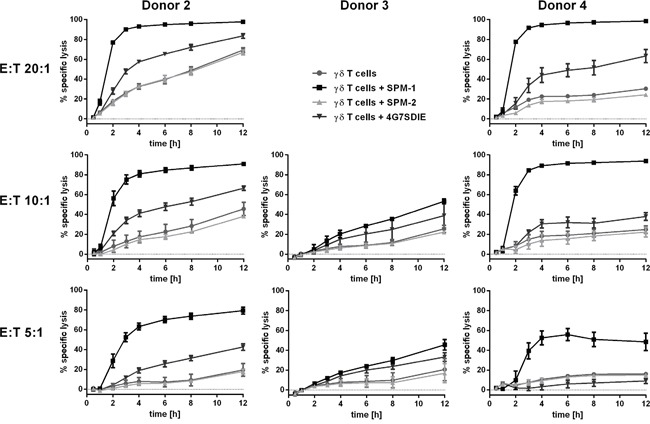
SPM-1 directs expanded γδ T cells from healthy donors for very rapid lysis of CD19-bearing MCF7-CD19 tm target cells, monitored in a real-time assay For γδ T cell donors # 2, 3 and 4 specific lysis curves were calculated from the cell indices (CI) of MCF7-CD19 tm cells, measured over the time course of the reaction with 1 nM SPM-1 or control proteins. SPM-2: control triplebody 33-16-123 with scFv binding domains for the target antigens CD33 and CD123, which are not carried by the MCF7-CD19 tm cells. CI values measured after treatment with control triplebody SPM-2 were comparable to those obtained with γδ T cell controls alone. Measurements were performed with the help of the label-free impedance-based xCelligence assay as described in Methods. Decreasing CI values are a correlate of and indicative of target cell lysis. In the presence of triplebody SPM-1, CI values decreased to approximately 9-fold lower values than after treatment with γδ T cells alone (Patient 4: 0.1 versus 0.9). Furthermore, specific lysis upon treatment with SPM-1 was more rapid and efficient than after treatment with ADCC-optimized CD19-antibody 4G7SDIE.

To assess whether γδ T cells are capable of lysing CD19-bearing target cells by ADCC in combination with SPM-1 or control agents, and to observe the progress of this reaction in real time, viability of CD19-expressing, adherent MCF7-CD19 tm cells was monitored with the xCelligence assay system [41, 42]. This assay was initially used here with freshly isolated, non-expanded γδ T cells from 2 healthy donors. The γδ T cells were enriched in one case by positive selection with immunomagnetic (MACS) beads and reached a purity of 97 %, but the absolute number of cells collected in this manner was low. Specific lysis was obtained in combination with both SPM-1 and the CD19 antibody 4G7SDIE, in particular at high E : T ratios of 10 : 1 and 20 : 1, but not with the control triplebody SPM-2 (data not shown). These results are consistent with earlier reports, in which specific lysis of MCF7-CD19 tm cells with similarly enriched non-expanded γδ T cells from healthy donors mediated by the 4G7SDIE antibody had been reported [41]. In conclusion, SPM-1 was capable of mediating ADCC of these adherent target cells by non-expanded γδ T cells from a healthy donor, but the available cell numbers were too low to permit a systematic study. Therefore, the experiments, which are described below (Figure 6; Supplementary Figure S1), were performed with *ex vivo* expanded γδ T cells.

For this purpose PBMCs from healthy volunteers obtained by leukapheresis were first expanded *ex vivo* in the presence of rhIL-2 and zolendronate as described previously [41]. After 12 -14 d in culture the total number of expanded cells was increased only marginally (by about 30 %), but the fraction of γδ T cells within the expanded population was increased by about 20 - 30-fold from typically 2 - 3 % to 35.9 – 60.6 %. The fraction of CD16-bearing γδ T cells in the expanded cell population was around 36 % for the sample from Donor 2. γδ T cells were then isolated from this population with immunomagnetic beads, which resulted in highly pure γδ T cells in sufficient numbers. The batch derived from Donor 2 was 98 % pure at this stage and was incubated once more overnight with IL-2 to remove contaminants resulting from the MACS enrichment procedure. This population was still heterogeneous with regard to CD16 antigen density on the cell surface. xCelligence assays were then performed with these cells using either SPM-1 or 4G7SDIE or control proteins as mediators of lysis. While the morphological and physiological properties of γδ T cells are likely to be affected by this *ex vivo* expansion, the use of this expanded population still permits us to conclude, whether γδ T cells as a defined T cell subset are capable in principle of achieving target cell lysis mediated by SPM-1.

Specific lysis values were calculated from the raw data and plotted for 3 different donors of γδ T cells at different E : T ratios as a function of time (Figure 6). Addition of SPM-1 caused a rapid increase in specific target cell lysis by γδ T cells from Donors 2 and 4 within the first 3 hrs. Close to 100 % of specific lysis was achieved within 12 hrs. By contrast, γδ T cells from Donor 3 did not produce a similarly rapid lysis during the first hours, but showed a steady slow increase in target cell lysis over the entire 12 hr measurement period. The control triplebody SPM-2 with binding sites for antigens CD33 and CD123 [27, 34], which were not expressed on these targets, did not cause target cell lysis beyond the spontaneous antibody-independent lysis (AIC) of this batch of γδ T cells alone (Figure 6). Lysis mediated by SPM-1 showed a faster initial rise than lysis mediated by the Fc-optimized antibody 4G7SDIE, although towards the end of the measurement period the reaction rates mediated by both agents appear to have stabilized and to have reached close to constant values. The raw data for Donor 4 from this experiment are also shown in Supplementary Figure S1, because this representation visualizes in a particularly clear manner that the lytic process as followed by this assay did not proceed with a mono-phasic, but with an at least bi-phasic kinetics. This observation suggests that at least 2 different so far unknown molecular processes are at work at different stages of the overall reaction. In the Discussion section we attempt to relate these results to the different molecular architecture of these antibody-derived agents.

## DISCUSSION

The key findings of this study are:

- The humanized and optimized triplebody SPM-1 can be produced in sufficient quantity and purity with an industry standard production and purification process, which can be scaled up for large-scale production.
- SPM-1 mediated efficient redirected lysis of CD19-bearing target cells in cell culture assays not only by NK cells but also by γδ T cells from healthy donors.
- In benchmark experiments with NK cells from healthy donors, SPM-1 was equally active as Fc-engineered antibody-derived proteins, including the CD19 antibody 4G7SDIE. It was active at similarly low EC_50_ concentrations in the picomolar range and mediated comparable maximum specific lysis as these best-in-class reference proteins.
- SPM-1 mediated efficient lysis of primary cells from patients with various B lineage malignancies and of cell lines derived from various B cell neoplasms by NK cells from healthy donors. The agent produced stronger ADCC lysis than Rituximab of primary cells from 3 B-CLL patients, one NHL patient and one MPAL (NOS) patient.
- Non-expanded γδ T cells from freshly drawn PBMC samples of healthy donors were activated for cytolysis by exposure to SPM-1 plus target cells, as evidenced by surface exposure of the degranulation marker CD107a and elevated intracellular concentrations of TNFα and IFN-γ.
- SPM-1 mediated lysis of surface-adherent target cells by both non-expanded and *ex vivo* expanded γδ T cells from healthy donors in the impedance-based xCelligence assay.
- In the xCelligence assay, SPM-1 mediated a rapid initial phase of lysis of surface-bound targets, faster than lysis mediated by the Fc-engineered reference antibody 4G7SDIE.

The humanized and optimized triplebody SPM-1 was equally potent in ADCC as the parental agent, and its production and purification by industry-standard procedures routinely used for commercial production of therapeutic antibodies was found to be possible. The ADCC assays presented here were performed with *ex vivo* expanded NK cells from healthy donors, which were pre-activated through long-term exposure to IL-2. We anticipate that SPM-1 will also be active in patients with B-lymphoid malignancies by directing autologous NK cells, provided it is administered at a time, when endogenous NK cells are present in sufficient numbers and are cytolytically active. This is the case for example for pediatric patients with acute lymphoblastic leukemia in a post-transplant setting a few weeks after transplantation, where donor-derived NK cells rapidly reconstitute the patient's blood and marrow and are active in mediating graft-versus-leukemia (GvL) effects [37]. It remains, however, to be demonstrated in experiments with autologous NK cells from patients not treated by a stem cell transplant, that these cells also achieve a sufficiently strong ADCC lysis of their cancer cells mediated by SPM-1. Corresponding studies have been performed for triplebody SPM-2, which mediated ADCC of a patient's AML cells by autologous NK cells, when these were drawn in first remission after a successful induction therapy [34]. The issue is still debated, because in AML autologous NK cells have been reported to be reduced in numbers and specific lytic potential in the tumor environment [19–22]. It is not clear, whether this reversible functional attenuation is equally important in B-lymphoid malignancies as in AML, because it is supposedly mediated at least in part by soluble mediators and the hypoxic milieu present in the marrow of AML patients. The tumor environment may be quite different for B-lymphoid malignancies, which evolve at different sites and in different cellular environments. We do not yet know, whether γδ T cells are present in sufficient numbers and in an active state for ADCC in patients with B cell malignancies at the sites of the cancer cells.

Triplebodies such as SPM-1 achieve similar cytotoxic effects as conventional therapeutic antibodies in cell culture ADCC assays in far lower concentrations. Rituximab for example is used in lymphoma therapy at concentrations in the range of 10 mg/kg, while Blinatumomab is used at concentrations in the range of 10 μg/kg, i.e. in approximately 1.000-fold lower doses [11]. In the results presented above with primary cells from patients with B cell malignancies (Figure 3), SPM-1 was active in ADCC assays in concert with NK cells with EC_50_-values lower by 5- to 430-fold than those determined for Rituximab. This finding is consistent with the dose ranges of Rituximab and Blinatumomab used in clinical applications as quoted above. The primary cells from the relapsed B-CLL patient, which were not efficiently lysed by NK cells plus Rituximab (Figure 3), were not antigen-loss variants and still expressed surface CD20. More likely, they were resistant to ADCC by NK cells through a different mechanism. Such cases have been reported for other Rituximab-resistant patients. However, this resistance cannot have been a general resistance to all different pathways leading to death of target cells by ADCC, because the cells were still lysed by ADCC via NK cells plus SPM-1.

The Fc-engineered CD19 antibody 4G7SDIE, which is largely identical with the corresponding antibody XmAb5574 [9, 39, 41], has previously been shown to mediate potent ADCC by γδ T cells [41]. This T cell subset is particularly useful in combination with TCRαβ- and CD19-depleted allogeneic stem cell transplantation (allo-HSCT) [47]. In such cases, both γδ T cells and NK cells are available for graft-versus-leukemia activity and can maintain a certain level of immune protection of the host. The γδ T cell population is small but potent, and does not only provide a natural anti-cancer activity, but is also unlikely to elicit graft-versus-host-disease (GvHD), because of its independence from MHC-restriction. Furthermore these cells mediate significant anti-viral activity. Therefore, γδ T cells are a highly desirable population of immune effector cells for cancer immunotherapy [37, 38]. The results presented here establish that the CD16-binding module carried in SPM-1 is capable of engaging both NK and γδ T cells as effectors for lysis of leukemia cells. Efficient activation and engagement of γδ T cells by SPM-1 was demonstrated here, and the potential expansion of this leukocyte subset *in vivo* in response to activation holds promise for the treatment of leukemia patients with triplebodies triggering γδ T cells via CD16.

The most unexpected new finding of the present study was produced with the help of the xCelligence assay, which allowed us to follow a cytolytic reaction mediated by a triplebody in real time. The following unresolved problem has intrigued us for several years: the parental triplebody ds(19-16-19) of SPM-1 showed an approximately 3-fold stronger binding avidity to CD19 on leukemic target cells than the bispecific tandem diabody 19-16, but had an approximately 25-fold greater cytolytic potential than this diabody for a number of malignant B-lymphoid cell lines and primary cell samples from a number of patients with different B-lymphoid cancers [26]. It was hypothesized that the triplebody may have led to the formation of a tighter synapse between the cancer cells and the NK cells, and that this in turn may have led to a stronger activation of the NK cells for cytolysis. However, no direct evidence in support of this hypothesis has been produced, because structural methods for a comparative analysis of the fine structure of the synapses produced by both agents were not available. It came as a surprise that progress towards solving this problem may come from improving the resolution of measurement methods for kinetic aspects of synapse formation and the cytolytic reaction.

The key observation reported here (Figure 6; Supplementary Figure S1) is that the cytolytic reaction, as followed by the xCelligence assay, occurred with a biphasic kinetic or a kinetic of even greater complexity. A rapid initial change in the cell index (CI), a measure of viability of the cells (equated with a rapid first phase of cellular lysis [42]), was followed by at least one more phase of slower viability changes. We do not know the precise correlation between the changes in the CI-value and cellular death, and whether a change in CI is only achieved when a target cell is completely lysed, or whether it also occurs, when a target cell is not yet irreversibly dead, but has engaged in the first steps of a multi-step pathway to death. Cell death mediated by NK cells through the degranulation of granzymes is death by apoptosis, and occurs through a succession of steps, which can be individually monitored. An example in case is the time-resolved study of apoptosis by cytofluorimetry with staining of the target cells by annexin V and propidium iodide [48]. In this case an early pre-apoptotic phase can be distinguished, characterized by annexin V staining but yet no influx of PI into the nucleus, which is still reversible. A subsequent late apoptotic state is characterized by more intense staining with both annexin V and PI, which is irreversible. It is not clear, whether the initial rapid change in the CI-value, which was observed here, reflects a truly irreversible step towards cellular death and complete lysis, mediated by γδ T cells, or only a change in impedance, which may be associated with reversible pre-apoptotic changes such as the rearrangements of the cellular membrane detected by binding of annexin V. Regardless of the precise correlation between events discovered by changes in CI in this assay and progressive stages on the path to cellular death as detected by other methods, the data shown here demonstrate the existence of more than one so far poorly understood reaction phases, which are likely to be highly informative about the mechanism of action of this triplebody.

A potential answer to the problem posed above, suggested by the results of the present study is that the strength of binding of a therapeutic protein to the target cell may not be the dominant determinant of its cytolytic potential. To build a productive cytolytic synapse, binding of the agent to the trigger on the effector cell is also important. Although the binding affinities of SPM-1 for CD16 on effector cells and of the CD16 receptor on effector cells for the Fc-domain of Fc-engineered antibodies such as 4G7SDIE are in the same order of magnitude, it is still possible that the triplebody has faster access to CD16 on the γδ T cell (and thus a faster “on”-rate) than the 4G7SDIE antibody. We suspect this to be the case, because the triplebody has only half the mass of the antibody and may have different space-filling properties and different flexibility, which may allow faster access to the CD16 epitope on the effector cell. This faster access may lead in turn to a faster formation of a synapse between the suface-adherent target cells and the triplebody-decorated γδ T cells. This could lead to the differences in the initial phase of changes in the CI index, which were detected by the xCelligence assay. This difference in the early stage of the kinetics may not affect the EC_50_-values and the degree of maximum specific lysis, because both SPM-1 and the 4G7SDIE antibody mediated target cell lysis by NK cells with similar EC_50_-values and similar maximum specific lysis (Figure 4B). It could however still have an impact on the pharmacokinetic properties of both agents, and thus on their anti-cancer activities *in vivo*. Therefore, the xCelligence assay offers a welcome enrichment of the set of tools available to study cytolytic processes in detail, even if it still does not allow for single cell resolution. Combining high throughput real-time assays with single-cell resolution assays is an important area for future improvements, which promises to be highly informative [49].

Taken together, the data presented here permit us to conclude that SPM-1 in combination with NK cells is highly active in ADCC reactions against primary cells from patients with a variety of B-lymphoid malignancies. It is capable of recruiting γδ T cells for cytolysis, and reveals unsuspected rapid reaction kinetics in time-resolved cytolysis assays. These properties make it both a valuable tool for further studies of the kinetics of the cytolytic process together with CD16-bearing effector cells, and a candidate for further clinical development as a highly potent alternative for the treatment of B-lymphoid malignancies with distinct advantages over available best-in-class agents.

## MATERIALS AND METHODS

### Generation of triplebodies and other antibodies and antibody-derived proteins

To produce triplebody SPM-1, the CD19-specific scFv domains contained in the parental triplebody ds(19-16-19) [26] were humanized with procedures developed in the laboratory of Dr. A. Honegger [43, 44]. The CD16-specific scFv was disulfide-stabilized according to published procedures [43]. SPM-1 contains a C-terminal hexa-histidine tag for purification purposes. Potentially immunogenic sequences that resulted from the standard cloning procedures used for the generation of the parental ds(19-16-19) were removed from the final protein. The coding sequence for SPM-1 was optimized to minimize homologous recombination between cDNA sequences coding for the CD19 binding domains by introducing variations in the wobble bases of the coding triplets. The cDNA was subcloned into a pSecTag2-HygroC expression vector (Life Technologies, Darmstadt, Germany). The Her2-16-Her2 triplebody was constructed by replacing the N- and C-terminal scFvs of a humanized successor molecule of the published triplebody ds(19-16-19) [26] with Her2-specific scFvs from the pSecTag2-HygroC-4D5-CD3 bispecific single chain Fv construct provided by Prof. M. Peipp [50] using standard molecular biology techniques.

The minibodies 4D5-IgG1-Fc and 4D5-IgG1-Fc engineered (provided by Prof. M. Peipp) contained a Her2 (clone 4D5)-specific scFv, a modified hinge region from an IgG, which allowed for disulfide-bridge formation, and a modified Fc-domain, harboring the substitutions S239D/I332E/A330L (SDIEAL) or no substitutions, respectively [50]. To generate the CD19-targeting minibodies, the 4D5-scFv was replaced by the CD19-targeting scFv from SPM-1.

For the expression of all proteins, human Freestyle^TM^ 293-F cells (Life Technologies, Darmstadt, Germany) were transfected with the respective plasmid DNA using the 293fectin^TM^ Transfection Reagent (Life Technologies, Darmstadt, Germany) according to the manufacturer's instructions. Proteins were either expressed transiently or a pool of stably transfected cells was generated by continuous selection with 50 μg/ml hygromycin C.

Recombinant proteins were captured from cell culture supernatants via their C-terminal hexahistidine tags by metal-ion affinity chromatography. SPM-1 was further purified by anion- and cation-exchange chromatography. The scFv-minibodies and Her2-16-Her2 were further purified by size exclusion chromatography. Protein concentrations were determined by absorbance measurements at 280 nm and using the molar extinction coefficient derived from the amino acid sequence.

The quality of purified recombinant protein batches was analyzed by analytical size exclusion chromatography to monitor the presence of monomeric protein, aggregates, breakdown products, incompletely synthesized products, contaminants and products generated by homologous recombination. An Äkta liquid chromatography system was used, equipped with a Superdex S200 5/150 GL column (GE Healthcare Europe, Munich, Germany). Total amounts of 25 μg of protein in 50 μl volumes were loaded onto the column followed by isocratic elution with SEC buffer (20 mM Histidine-HCl pH 6.0, 150 mM NaCl). Eluted proteins were monitored by absorbance at 280 nm and by SDS-PAGE.

The CD19 antibody 4G7SDIE was provided by Prof. G. Jung and Dr. L. Grosse-Hovest from the University of Tübingen, Germany. This antibody harbors an engineered Fc-domain with the same substitutions S239D and I332E as the CD19 antibody XmAb 5574 [9, 39, 41]. The therapeutic CD20 antibody Rituximab (MabThera®) was obtained from Roche Pharma AG.

### Target cells and culture conditions

The RAJI, NALM-6, SEM, NAMALWA, ARH-77 and MCF-7 cell lines were obtained from the German Collection of Microorganisms and Cell Cultures (DSMZ, Braunschweig, Germany). RAJI, NALM-6, NAMALWA and ARH-77 were cultured in RPMI 1640 medium (Invitrogen, Karlsruhe, Germany). MCF-7 and SEM cells were kept in EMEM and IMDM medium (Lonza, Basel, Switzerland), respectively. All media were supplemented with 10 % fetal calf serum or pooled human AB serum (Invitrogen, Karlsruhe, Germany), 100 U/ml penicillin, 100 μg/ml streptomycin, 1 mM sodium pyruvate and 2 mM L-glutamine (all reagents from Biochrome).

MCF-7-CD19 tm cells were generated as described [41]. Briefly, full-length cDNA coding for human CD19 (GenBank BC006338.2) was purchased from Source Bioscience (Berlin, Germany) and cloned into a suitable cDNA expression vector. MCF-7 cells were transfected with this plasmid by electroporation, and CD19-expressing clones were selected and sorted by flow cytometric analysis, respectively.

### Preparation of primary cells from blood of human donors

Peripheral blood samples were drawn from subjects into EDTA solution at the Medical Center of the LMU Munich or isolated from platelet-pheresis products provided by the Institute for Transfusion Medicine of the University of Tübingen after receiving informed written consent. The project was approved by the Ethics Committee of the University of Munich Medical Center. Mononuclear cells (MNCs) from leukemia patients and healthy donors were enriched by density gradient centrifugation using the Lymphoflot reagent (Biotest, Dreieich, Germany) or Biocol Separating Solution (Biochrom, Berlin, Germany) according to manufacturer's instructions. PBMCs, also used as the source of leukemia cells from patient samples, were then either suspended in RPMI medium (Life Technologies) containing 10 % fetal bovine serum (FBS) with penicillin and streptomycin (PS) at 100 units/ml and 100 μg/ml, respectively, for immediate use, or stored frozen in a solution containing 90 % FBS and 10 % DMSO. Cell viability was assessed by Trypan blue exclusion before use.

### *Ex vivo* expansion of MNCs as a source of NK cells from healthy donors

PBMCs were expanded *ex vivo* in RPMI medium containing Interleukin-2 (IL-2) plus 5 % human serum (Life Technologies) for 20 d after an initial period of culture in the presence of an OKT3 (CD3) antibody, and were then frozen in aliquots for subsequent use as described [30, 51]. Prior to use in cytolysis experiments, the cells were thawed and cultured overnight in RPMI medium containing 5 % human serum plus 50 units/ml and 50 μg/ml PS, respectively, but no additional IL-2.

### Expansion of γδ T cells

PBMCs were seeded at 1.5 x 10^6^ cells per well in 6-well plates and cultured in supplemented IMDM medium containing 100 IU/ml of recombinant human IL-2 (rhIL-2) (Novartis, Basel, Switzerland) and 400 nM zolendronate (Hexal, Holzkirchen, Germany). After 13 - 14 d of culture, expanded populations containing 35.9 - 60.6 % of γδ T cells were positively selected using a Hapten-modified TCR-γδ antibody and FITC-conjugated anti-Hapten MicroBeads with the autoMACS system (Miltenyi, Bergisch Gladbach, Germany). Purity of the isolated populations was (99.2 ± 0.8) % (n = 3) and isolated cells were incubated with 400 IU/ml rhIL-2 overnight prior to functional assays. Isolated γδ T cells had lost their FITC-labeling and had restored surface expression of TCR-γδ after 24 hrs (U Seidel, unpublished data).

### Flow cytometric analysis

Flow cytometric analysis was performed with an Accuri C6 flow cytometer (BD Biosciences, Heidelberg, Germany). CD16- and CD56-specific monoclonal antibodies (mAbs) were used for the analysis of NK cell content in PBMC-preparations and measured against isotype control mAbs (Immunotech, Marseille, France). Unlabelled CD19- and CD20-specific and isotype control mAbs (BD Pharmingen) were used for the analysis of cell surface densities of the antigens on target cells. Surface expression was measured using a calibrated cytofluorimetric assay (QIFI KIT^®^; DAKO; Hamburg, Germany) as described [45, 52].

### Determination of equilibrium binding constants (K_D_) and serum stability measurements of SPM-1

Equilibrium binding constants (K_D_ values) were measured using a flow cytometry method as previously described [46]. The K_D_ values were calculated from the raw data by using a nonlinear regression curve fit with the help of GraphPad Prism 3 (Graph Pad Software, Inc, San Diego, CA). Measurement of *in vitro* stability of SPM-1 in human serum was performed as previously described [26].

### Redirected lysis (RDL) assays using calcein release

Non-radioactive cytolysis assays based on the release of calcein from target cells were performed as previously described [30]. Calcein AM (Life Technologies)-labeled target cells were mixed with effector cells (*ex vivo* expanded MNCs) in RPMI 1640 GlutaMAX medium supplemented with 10 % FCS and 1 % Penicillin/Streptomycin at an E (NK cell) : T ratio of 2 : 1. After addition of different concentrations of SPM-1 or control antibodies and antibody derivates (minibodies), respectively, reactions were incubated at 37°C with 5 % CO_2_ for 3 - 4 hrs. Calcein release was then determined by measuring the fluorescence intensity (relative light units, RLU) in the supernatant with a Tecan Infinite M1000 microplate reader (Tecan Group Ltd, Männedorf) at 485/535 nm. Maximum lysis was achieved by addition 2.5 % Triton X-100. Specific lysis was calculated as follows:

$$\%specific\;Lysis=100*\frac{\left[RLU(sample)-RLU(background\;release)\right]}{RLU(\max lysis)-RLU(background)]}$$

EC_50_-values (concentration of triplebody producing 50 % of maximum specific lysis) were determined using sigmoidal dose-response curve fits (GraphPadPrism, San Diego, CA).

### γδ T cell degranulation assay using detection of cell surface CD107a

The fraction of γδ T cells (CD3^+^, TCRγδ^+^) was determined by flow cytometry. Samples with γδ T cell counts above 1.5 % were selected for CD107a assays. Equal numbers of PBMC and NALM-6 or SEM cells were incubated with 1 nM SPM-1 or control triplebody Her2-16-Her2, 2 μM GolgiStop reagent (BD Biosciences), 10 μg/ml Brefeldin A (Sigma, Steinheim, Germany) and the fluorescent labeled CD107a-APC antibody (Biolegend, San Diego, USA) overnight in supplemented IMDM medium at 37°C in an incubator with a 5 % CO_2_-atmosphere. PBMCs were then stained for surface and intracellular markers and analyzed by flow cytometry.

### Intracellular cytokine staining (ICS) of γδ T cells for TNFα and IFN-γ

Intracellular cytokine staining of γδ T cells was performed with IFNγ-BV711, TNFα-PB, and isotype control antibodies supplied by Biolegend (San Diego, CA, USA). Briefly, after incubation of γδ T cells with SEM or NALM-6 cells and SPM-1 triplebody or controls, cells were washed and resuspended in 100 μl of PBS : 1 % albumin solution. Cells were then stained with γδ TCR mAb (BD Biosciences, Heidelberg, Germany) for 30 min on ice. To distinguish TNFα and IFN-γ producers within the γδ T cell populations, cells were washed, fixed and permeabilized by incubation with 250 μl of Cytofix/Cytoperm solution (BD Biosciences, Heidelberg, Germany) for 15 min. Cells were then stained with either PE conjugated TNFα or IFN-γ mAbs versus a control mAb for 30 min on ice. The cells were finally washed twice, resuspended in FACS buffer (PBS containing 2 % fetal calf serum (FCS) plus 0.05 % sodium azide) and analyzed by flow cytometry. The percentage of cytokine-positive cells within the given γδ T cell treatment group was then calculated and plotted.

### Impedance-based cytotolysis assay with human γδ T cells as cytolytic effectors

The cytolytic potential of expanded γδ T cells was analyzed in a real-time cytotoxicty assay with an xCelligence RTCA SP instrument (ACEA Biosciences, San Diego, CA) as previously described [41, 42]. Briefly, 5x10^3^ MCF-7-CD19 tm cells were seeded into each well. Expanded γδ T cells and 1 nM SPM-1 or control triplebody Her2-16-Her2, respectively, were added 24 hrs later. Cell viability was monitored every 15 min for 48 hrs. Cell indexes (CI) were normalized to CI of the time-point, when γδ T cells were added, and specific lysis was calculated relative to control cells without any added γδ T cells.

## SUPPLEMENTARY FIGURE


