# Supplementary material for: CD19-specific triplebody SPM-1 engages NK and γδ T cells for rapid and efficient lysis of malignant B-lymphoid cells
Source: Oncotarget. 2016 Nov 4;7(50):83392–408. doi: 10.18632/oncotarget.13110 (PMC5347777; doi:10.18632/oncotarget.13110)
Supplement: Supplementary file 1 [file oncotarget-07-83392-s001.pdf]

CD19-specific triplebody SPM-1 engages NK and γδ T cells for rapid and efficient lysis of malignant B-lymphoid cells

SUPPLEMENTARY FIGURE

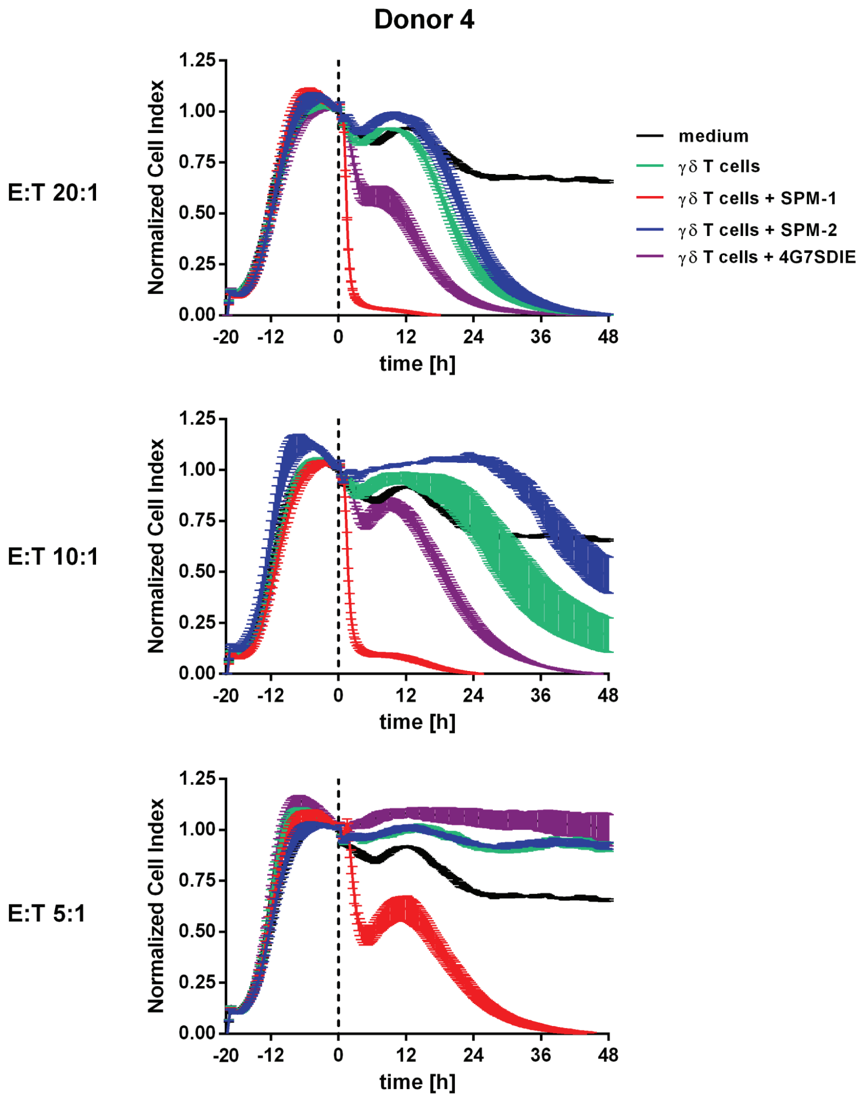

Supplementary Figure S1: SPM-1 directs expanded γδ T cells from healthy donors for very rapid lysis of CD19-bearing MCF7-CD19 tm target cells, monitored in a real-time assay. Raw data for the processed data of Donor 4 shown in Fig. 6. Cell indexes (CI) plotted here are a measure of the fraction of living cells contained in a population at the time of measurement. Adherent CD19-bearing MCF7-CD19 tm target cells were allowed to form a layer on the chip. Time of addition of the effector cells plus mediator protein (t_0) is indicated by a vertical dashed line. Normalized cell indices (CI) were measured as described in Methods and are a close correlate of life cells on the chip. SPM-1 caused a substantially more rapid loss in viability of the target cells than the best-in-class CD19-antibody 4G7SDIE, and the kinetics of loss of viability was not mono-phasic but showed clear evidence for at least 2 phases of the reaction, as evidenced by the shoulders and secondary peaks in the reaction profile.
